# A Bi-layer Composite Film Based on TiO_2_ Hollow Spheres, P25, and Multi-walled Carbon Nanotubes for Efficient Photoanode of Dye-sensitized Solar Cell

**DOI:** 10.1007/s40820-015-0081-1

**Published:** 2016-02-02

**Authors:** Putao Zhang, Zhiqiang Hu, Yan Wang, Yiying Qin, Wenqin Li, Jinmin Wang

**Affiliations:** 1 Institute of New Energy Material, Dalian Polytechnic University, Dalian, 116034 People’s Republic of China; 2 School of Environmental and Materials Engineering, College of Engineering, Shanghai Second Polytechnic University, Shanghai, 201209 People’s Republic of China

**Keywords:** Dye-sensitized solar cell, TiO_2_, Hollow spheres, Carbon nanotubes

## Abstract

**Abstract:**

A bi-layer photoanode for dye-sensitized solar cell (DSSC) was fabricated, in which TiO_2_ hollow spheres (THSs) were designed as a scattering layer and P25/multi-walled carbon nanotubes (MWNTs) as an under-layer. The THSs were synthesized by a sacrifice template method and showed good light scattering ability as an over-layer of the photoanode. MWNTs were mixed with P25 to form an under-layer of the photoanode to improve the electron transmission ability of the photoanode. The power conversion efficiency of this kind of DSSC with bi-layer was enhanced to 5.13 %, which is 14.25 % higher than that of pure P25 DSSC.

**Graphical Abstract:**

A bi-layer composite photoanode based on P25/MWNTs-THSs with improved light scattering and electron transmission, which will provide a new insight into fabrication and structure design of highly efficient dye-sensitized solar cells.
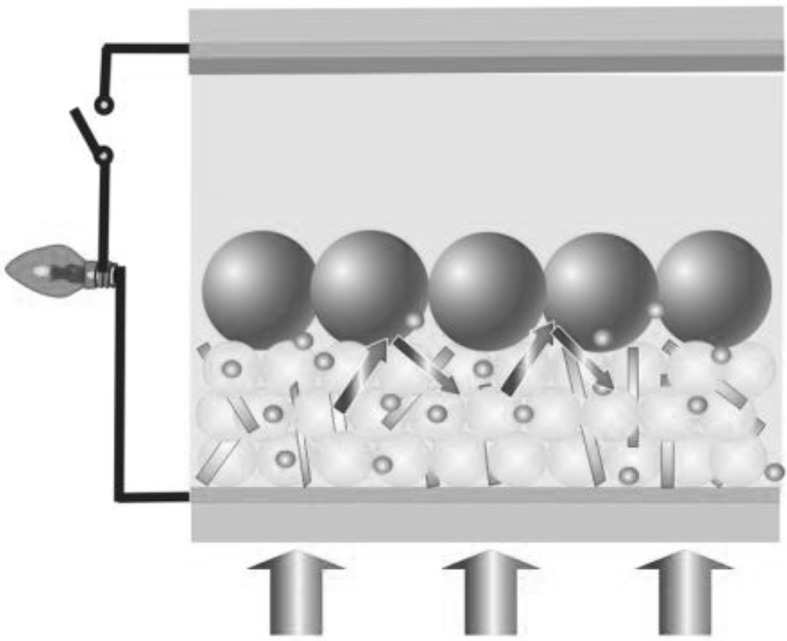

## Introduction

In the past two decades, dye-sensitized solar cells (DSSCs) have attracted extensive attention due to their low cost, convenient fabrication, and less pollution to the environment [1, 2]. As one critical part of a DSSC, photoanode is usually made of nanocrystalline TiO_2_ screen printed onto fluorine-doped tin oxide (FTO) substrate. A photoanode with high performance should have excellent light absorption ability and effective electron transmission ability. However, these two features usually negate each other for single-layer nanocrystalline photoanode films. So, two or more layers of TiO_2_ nanostructures are used to prepare complex nanocrystalline photoanodes, which is expected to having superior photovoltaic performance [3].

To improve the power conversion efficiency of DSSCs, researchers have made extensive efforts to modify the photoanodes [4–14]. In order to increase the absorption of sunlight and improve the photoelectric performance, scattering layers have been studied extensively. A variety of TiO_2_ nanostructures [15–19] have been used as scattering layers. The introduction of these nanostructures increases the light propagation path in photoanodes, resulting in increased light absorption of DSSCs. Wang et al. prepared TiO_2_ hollow spheres embedded with SnO_2_ nanobeans as the scattering layer, which shows an efficient scattering effect [20]. Xiong et al. introduced titania films with hierarchical structure as scattering layers [21], and Shi et al. prepared hollow TiO_2_ boxes as the scattering layer [22]. However, only increasing the absorption of light is not enough to improve the photovoltaic performance of DSSCs, researchers try to improve electronic transmission in photoanodes to improve the photovoltaic performance of DSSCs. One-dimensional (1D) TiO_2_ nanostructures such as nanorods, nanowires, nanobelts, and nanotubes have been used in photoanodes with excellent electron transport ability [23–30]. Although the introduction of 1D TiO_2_ nanostructures accelerates the electron transmission, the absorption of light is not improved at the same time. Hence, both electron transmission and light absorption should be considered to achieve a high-performance DSSC.

In this work, for light absorption, TiO_2_ hollow spheres were synthesized and used as the scattering layer; for electron transmission, multi-walled carbon nanotubes (MWNTs) were mixed with P25 and used as the under-layer. Thus, a bi-layer composite film containing TiO_2_ hollow spheres (THSs), P25, and MWNTs were used as the photoanode of a DSSC, exhibiting improved power conversion efficiency.

## Experimental Section

### Materials

Ethanol, α-terpineol, concentrated sulfuric acid, ethyl cellulose, acetylacetone, octylphenol polyoxyethylene ether (OP-10) emulsifiers, and tetrabutyl titanate (TBT) were purchased from Sinopharm Chemical Reagent Co. Ltd. and used without any further purification. MWNTs were purchased from Shanghai Lark Chemical Technology Co. Ltd. P25 was purchased from Dalian Qiseguang Solar Technology Development Co. Ltd.

### Synthesis of THSs

Monodisperse polystyrene (PS) spheres were used as a template, which were prepared via a boiling emulsifier-free emulsion polymerization according to pervious work [31]. In a typical preparation procedure, 2.0 g of PS spheres were placed into 20 mL of concentrated sulfuric acid under vigorous magnetic stirring at 40 °C for 15 h. After centrifugation and washing with ethanol for three times, sulfonated polystyrene (sPS) spheres were formed. Then 1.0 mL of tetrabutyl titanate and 15 mL of ethanol were mixed, and 1.0 g of sPS spheres were put into this mixed solution under magnetic stirring at room temperature for 1 h. After centrifugation and drying at 60 °C for 12 h, sPS spheres were coated with tetrabutyl titanate (tPS). The tPS spheres were calcined at 300 °C for 2 h in air to remove sPS spheres. To obtain THSs with the desired crystallinity, the powders were calcined in air at 500 °C for 30 min.

### Pretreatment of MWNTs

MWNTs were pretreated with mixed acid according to previous work [32]. In a typical process, 0.5 g of MWNTs were oxidized in 80 mL of 1:3 (V:V) concentrated nitric acid–sulfuric acid mixed solution under ultrasonication at 60 °C for 10 h. Then, the MWNTs were separated by centrifugation and washed with distilled water several times until a final pH value of 6. The product was subsequently dried in a vacuum oven at 40 °C for 12 h.

### Fabrication of DSSCs

**Fig. 1:**
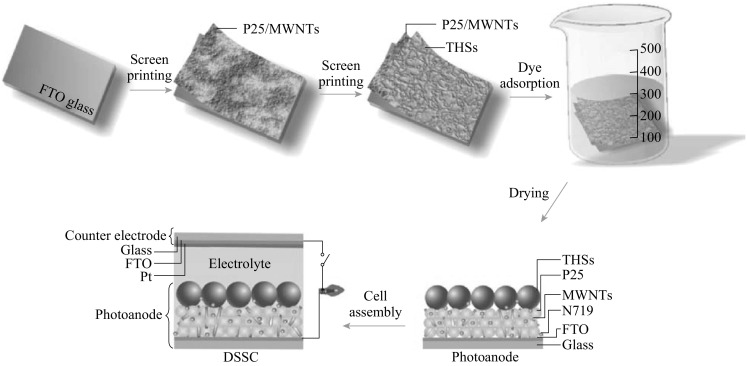
Schematic illustration for the preparation of P25/MWNTs-THSs DSSC

For the preparation of the P25 paste, 1.0 g of P25, 10 mL of ethanol, 0.8 g of ethyl cellulose, 4.3 mL of α-terpineol, 0.3 mL of acetylacetone, and 2 drops of OP-10 emulsifiers were mixed and grinded in an agate mortar for 1 h. THS paste and P25/MWNT paste (MWNTs, 0.1 wt%) were prepared by the same way [33]. To fabricate photoanodes, FTO-coated glasses were used as substrates, which were cleaned by sonication in acetone, deionized water, and ethanol each for 15 min, then blow dried with N_2_. TiO_2_ films were prepared by screen printing method. The film thickness was controlled by screen printing times. The films were heated at 325, 375, and 425 °C each for 5 min, 450 °C for 15 min, and at 500 °C for 30 min. After thermal treatment, the films were cooled down to 80 °C for dye sensitization for 24 h, then rinsed with ethanol and dried. The dye solution was 0.5 mmol L^−1^ N719 acetonitrile/tertiary butanol (V/V = 1:1) solution. Counter electrodes were prepared by spin-coating of 0.02 mol L^−1^ H_2_PtCl_6_ isopropyl alcohol solution onto FTO glass, and then heating at 400 °C for 15 min. Finally, the sensitized photoanode was sealed together with the counter electrode, followed by the injection of electrolyte solution. The electrolyte solution is composed of 0.05 mol L^−1^ I_2_, 0.10 mol L^−1^ LiI, 0.60 mol L^−1^ N-methyl-N-butyl imidazolium iodide (BMII), and 0.50 mol L^−1^ 4-tert-butylpyridine (TBP) in acetonitrile. The fabrication process of the photoanode is illustrated in Fig. 1.

## Characterization

The morphology of the sample was observed by field-emission scanning electron microscopy (FESEM, S-4800; Shimadzu Corporation). X-ray diffraction (XRD) pattern was used to study the crystal structure of the samples, using Cu *K*_α_ (*λ* = 0.15418 nm) radiation in the 2*θ* range from 10° to 70° with a scanning speed of 10° min^−1^. The UV–Visible absorption spectrum was obtained by a UV–Visible spectrophotometer (UV-2600, Shimadzu Corporation). The current–voltage (*J*–*V*) characteristics and electrochemical impedance spectra (EIS) of the DSSCs were measured by an electrochemical workstation (Autolab, PGSTAT302 N, Metrohm) under a light source for AM 1.5 radiation (Sun 2000 Solar Simulator, Abet Technologies).

## Results and Discussion

**Fig. 2:**
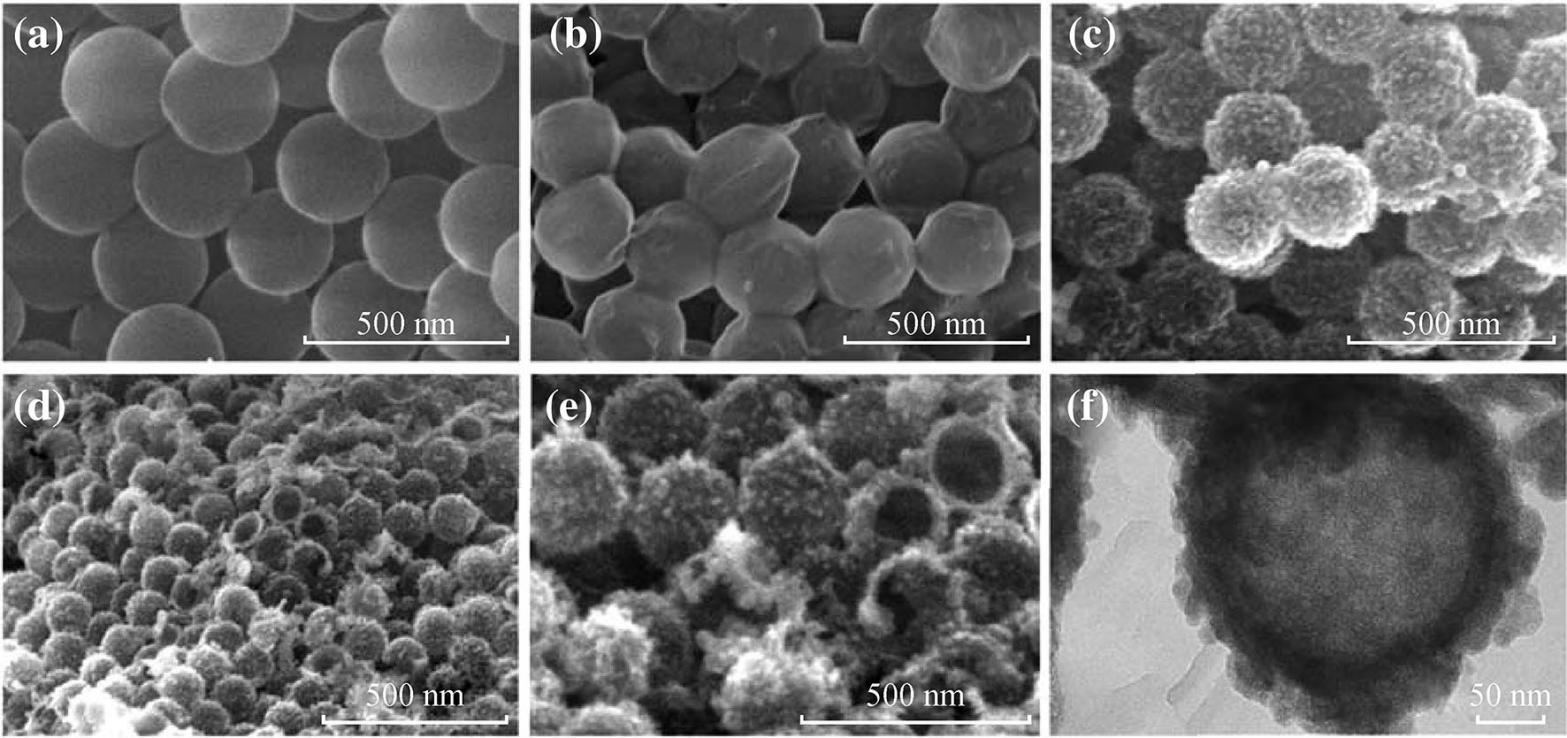
FESEM images of **a** PS, **b** sulfonated PS, **c** PS coated by tetrabutyl titanate, and **d**, **e** THSs, and **f** TEM image of a single THS

Figure 2 shows the FESEM images of PS spheres, sulfonated PS spheres, PS spheres coated by tetrabutyl titanate, THSs, and the TEM image of a single THS. It can be seen from Fig. 2a that the well-dispersed PS spheres with a uniform diameter of ~250 nm and smooth surface were synthesized. After being sulfonated by concentrated sulfuric acid, the PS spheres were converted to sPS (Fig. 2b). Obviously, the surface of sPS is rougher than that of PS and the shape of sPS turns into irregular spheres. We can see some sPS spheres adhering together. Using tetrabutyl titanate (TBT) as a titania precursor to coat the sPS, the result shows that TBT is easily adsorbed onto the surface of sPS because of containing a large number of negative ions. The surface of tetrabutyl titanate-coated sPS spheres (with a diameter of ~300 nm) is very rough. From Fig. 2d, we can see that some broken THSs, which indicates that the template has been successfully removed. We can obtain the outer diameters and wall thickness of the THS from Fig. 2e. The two adjacent broken THSs have a two-wall thickness of ~50 nm, so the wall thickness of a THS is ~25 nm. The TEM image of a single THS (Fig. 2f) further confirms the existence of hollow cavity in the as-prepared product. The measured wall thickness is ~25 nm, and the surface is very rough. This corresponds well with the FESEM observations.

**Fig. 3:**
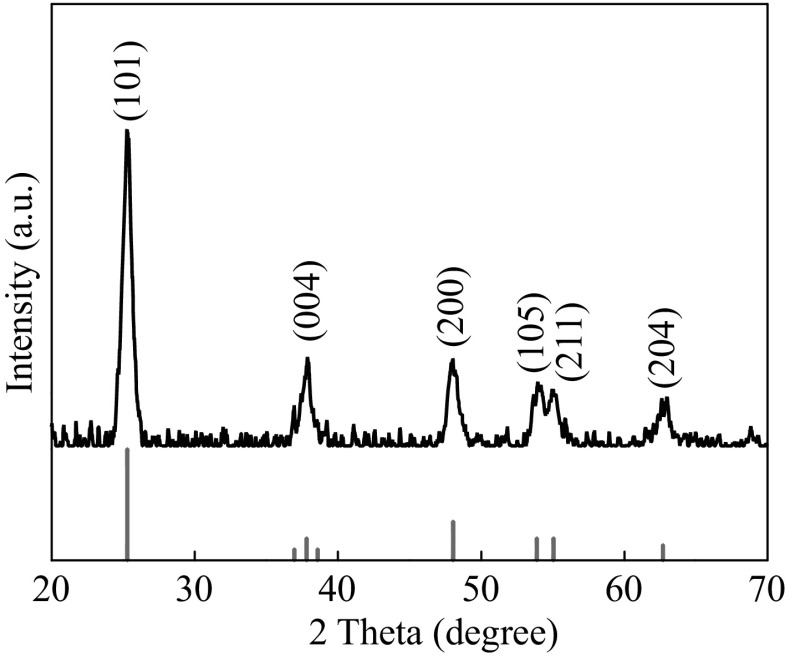
XRD pattern of the as-prepared THSs at 500 °C for 30 min

The XRD pattern of the as-prepared THSs is shown in Fig. 3. All the sharp peaks are in good agreement with the standard card of anatase TiO_2_ (JCPDS No. 21-1272). The sample is well crystalline and without any other impurities.

**Fig. 4:**
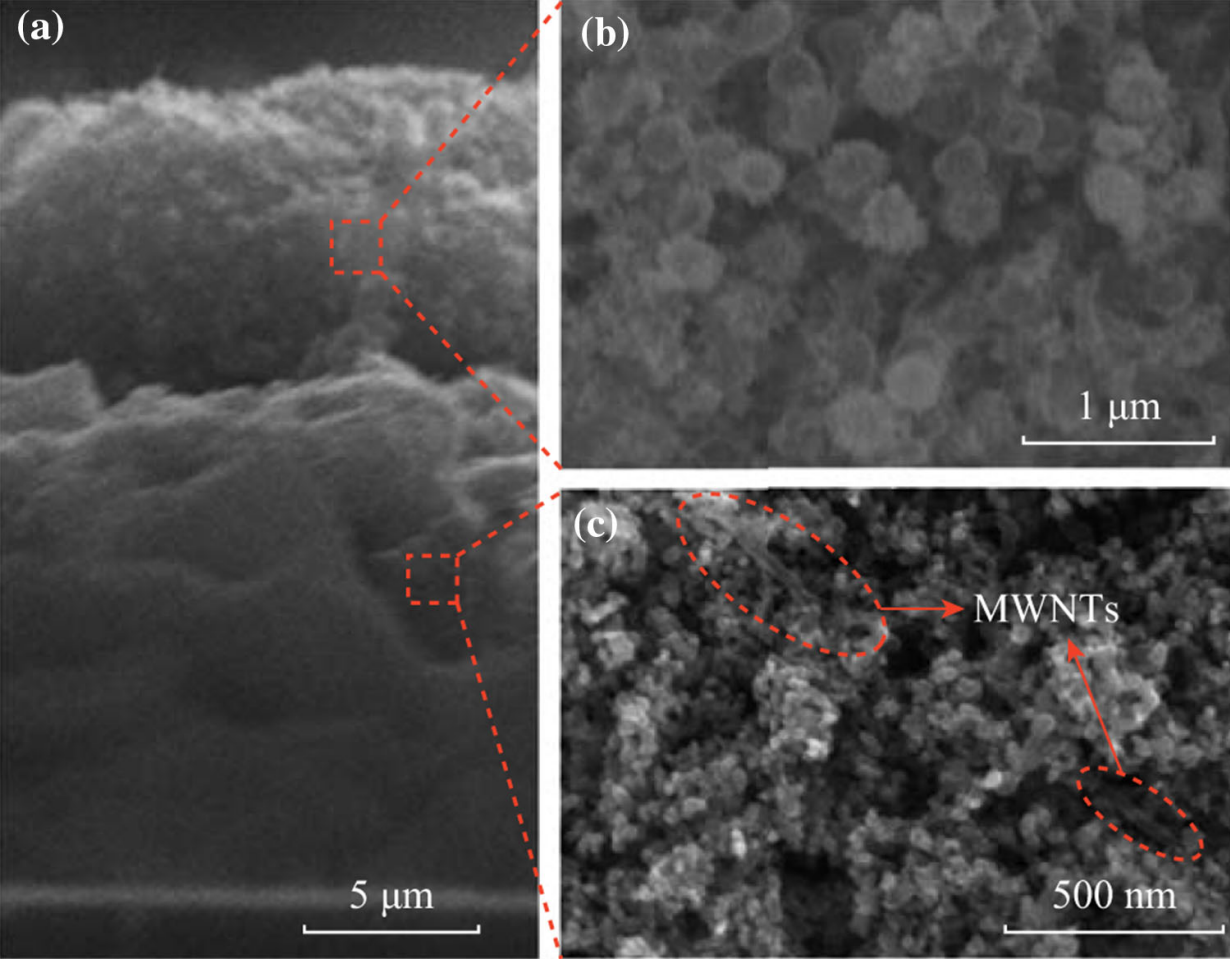
FESEM images of **a** cross section, **b** scattering layer containing THSs, and **c** under-layer containing P25/MWNTs

Figure 4a shows the cross-sectional FESEM image of the P25/MWNTs-THSs bi-layer film. It is seen that the thickness of the under-layer and the scattering layer is ~15 and ~8 μm, respectively. Under high magnification, the scattering layer (see Fig. 4b) and the under-layer (see Fig. 4c) can be observed more clearly. The scattering layer containing THSs is composed of a disordered macroporous network, and these macropores in the scattering layer can enhance the absorption of light and the transfer of electrolyte [34–36]. The under-layer consisting of P25 and MWNTs can be seen in Fig. 4c. Some MWNTs are embedded in P25, which can enhance the electron transport of photoanode [32].

**Fig. 5:**
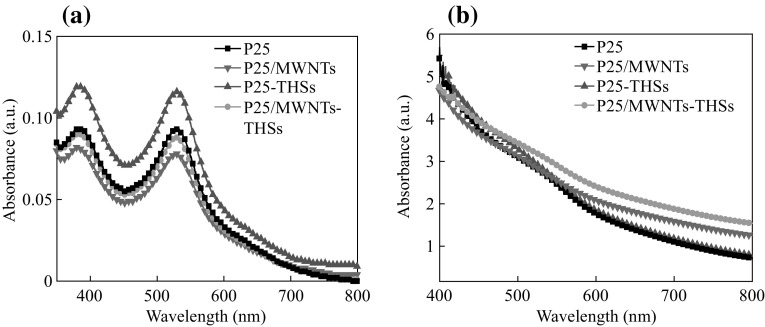
Absorption spectra of dye molecules desorbed from the dye-sensitized photoanodes (**a**) and absorption spectra of dye-sensitized films (**b**) of P25, P25/MWNTs, P25-THSs, and P25/MWNTs-THSs

The dye molecules were desorbed from the dye-sensitized photoanodes of P25, P25/MWNTs, P25-THSs, and P25/MWNTs-THSs, and the corresponding UV–Vis absorbance spectra are shown in Fig. 5a. From these spectra, it can be seen that the absorbance of dye desorbed from the P25/THSs photoanode is higher than that of other photoanodes, P25 and P25/MWNTs-THSs nearly equal, and P25/MWNTs slightly lower. The specific values of dye molecules adsorbed in photoanodes can be calculated, the calculated amounts of desorbed dye are 1.53 × 10^−7^, 1.23 × 10^−7^, 1.16 × 10^−7^, and 1.03 × 10^−7^ mol cm^−2^ for P25-THSs, P25, P25/MWNTs-THSs, and P25/MWNTs photoanodes, respectively. The N719 dye was desorbed from the TiO_2_ films with 0.2 mol L^−1^ NaOH aqueous solution (5 mL), and the UV–Vis absorption spectra (Fig. 5a) of the obtained solution were measured. The concentration of N719 in NaOH aqueous solution (*c*) can be calculated by the equation *A* = *Kcl*, where *l* is the path length of the light beam, *K* is the molar extinction coefficient of N719 at 515 nm, and *A* is the intensity of UV–Vis absorption spectra at 515 nm [37]. The obtained concentration multiplied by the volume of NaOH aqueous solution is equal to the amount of adsorbed N719, thus dye adsorption densities can be obtained accordingly. Figure 5b shows the absorption spectra of dye-sensitized films. Compared with the films of P25 and P25/MWNTs, P25-THSs and P25/MWNTs-THSs films have better absorption capacity in the wavelength range from 420 to 550 nm, which can be ascribed to the introduction of THSs as the scattering layer, and more light is reflected from the THSs layer and re-adsorbed by the under-layer. The films of P25/MWNTs and P25/MWNTs-THSs show enhanced absorption at higher range (>550 nm), because MWNTs can absorb some sunlight [38].

**Fig. 6:**
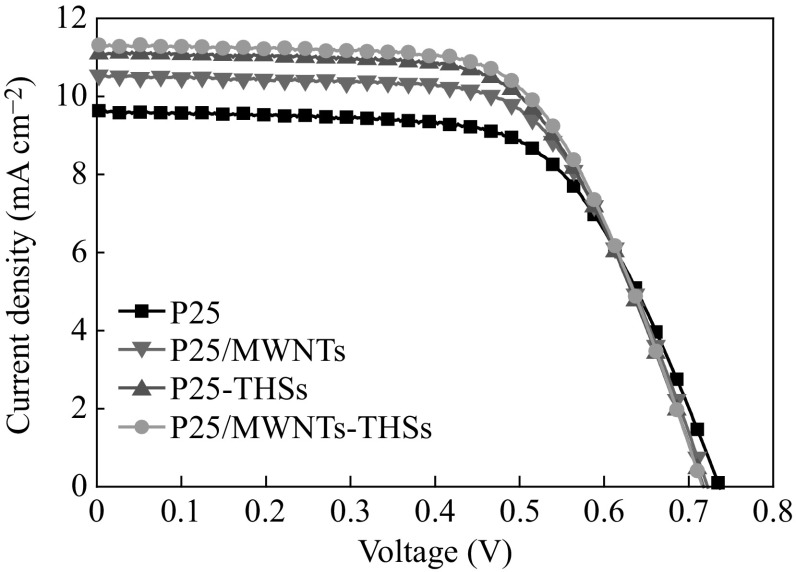
*J*–*V* curves of four kinds of DSSCs

**Fig. 7:**
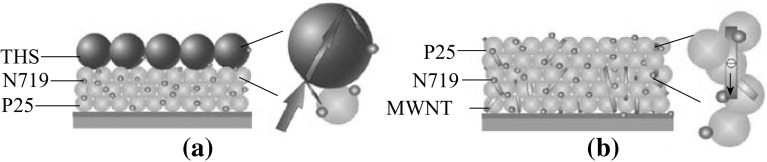
Schematic illustration of photoanodes based on (**a**) P25-THSs and (**b**) P25/MWNTs, showing different light scattering and electron transport effects

Performances of DSSCs with the four kinds of photoanodes are examined under one sun AM 1.5 radiation simulated sunlight. Figure 6 presents the *J*–*V* curves for the four kinds of solar cells. The corresponding photovoltaic parameters are summarized in Table 1. The DSSCs assembled with P25/MWNTs-THSs photoanode obtain an open voltage (*V*_oc_) of 0.72 V, a short circuit density (*J*_sc_) of 11.31 mA cm^−2^, a fill factor (*FF*) of 0.63, and a conversion efficiency (*η*) of 5.13 %. It is interesting to note that *J*_sc_ and *η* vary significantly from 9.63 (pure P25) to 11.31 mA cm^−2^ (P25/MWNTs-THSs), and 4.49 (pure P25) to 5.13 % (P25/MWNTs-THSs), respectively, compared to the DSSC based on pure P25 photoanode. The highly improved *J*_sc_ and *η* can be mainly attributed to two reasons: (a) the enhanced light harvesting due to the strong light scattering ability of THSs and (b) the improved electron transmission performance from the introduced MWNTs. The two reasons can be illustrated through comparing the photovoltaic properties of P25-THSs and P25/MWNTs solar cells with pure P25 solar cell. It is noted that the *V*_oc_ values (Table 1) of the four types of cells are not compatible with dye adsorption density. *V*_oc_ value is determined by many factors, such as recombination resistance, dye adsorption density, and surface morphology of nanocrystalline photoanode [39, 40]. As shown in Fig. 7a, when P25 nanoparticles and THSs are used as the under-layer and over-layer, respectively, the THSs play a key role in increasing the light scattering. More light is reflected from the THSs layer and re-adsorbed by P25 nanoparticle layer, which is beneficial to light harvesting and improving the power conversion efficiency of DSSCs (*η*_P25_ = 4.49 %, *η*_P25-THSs_ = 4.95 %). As shown in Fig. 7b, composite photoanode based on P25/MWNTs, due to the direct transport pathways provided by MWNTs, the electron transport rate is increased, and hence the charge recombination is reduced to some extent. This is also one of the factors to improve the power conversion efficiency of DSSCs (*η*_P25_ = 4.49 %, *η*_P25/MWNTs_ = 4.85 %). When putting THSs and MWNTs together into one photoanode, it exhibits an improved power conversion efficiency (*η* = 5.13 %).

**Table 1:** Photovoltaic properties of DSSCs with different photoanodes

DSSC type	*V*_oc_ (V)	*J*_sc_ (mA cm^−2^)	*FF*	*η* (%)
P25	0.74	9.63	0.63	4.49
P25/MWNTs	0.73	10.54	0.63	4.85
P25-THSs	0.72	11.08	0.62	4.95
P25/MWNTs-THSs	0.72	11.31	0.63	5.13

**Fig. 8:**
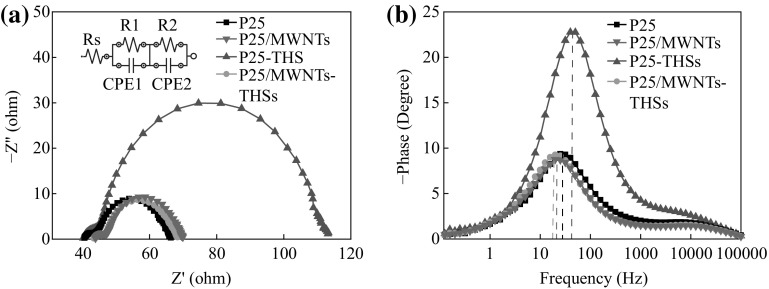
**a** Nyquist plot and **b** Bolt plot of DSSCs based on P25, P25/MWNTs, P25-THSs, and P25/MWNTs-THSs photoanodes. The *inset* in Fig. 8a displays the equivalent circuit model

To deeply understand the effect of THSs and MWNTs on the performance of DSSCs, and hence reveal the electron transport within the DSSCs, electrochemical impedance spectroscopy (EIS) is further studied. EIS spectra of the four kinds of cells were observed under the illumination of one sun (AM 1.5, 100 mW cm^−2^) at the open circuit. The results of four samples are shown in the Nyquist plots (Fig. 8a) and the Bode curves (Fig. 8b). From Fig. 8a, we can see that the spectra are composed of two semicircles situated in high and middle frequency regimes. The small semicircle in the high-frequency region is related to charge transfer resistance (*R*_1_) and constant phase element (CPE1) at the electrolyte/Pt counter electrode interface. The large semicircle in the middle frequency region is related to recombination resistance (*R*_2_) and constant phase element (CPE2) at the TiO_2_/dye/electrolyte interface. The inset of Fig. 8a is an equivalent circuit model, and the specific data of each element are listed in Table 2. We can see obviously that *R*_1_ is changed slightly and *R*_2_ shows a great difference. *R*_1_ is changed slightly because the four kinds of cells use the same Pt counter electrode and the same electrolyte, and *R*_1_ is related to the charge transfer at the interface between the electrolyte and the counter electrode. For recombination resistance, *R*_2_ noticeably increases (from 21.06 to 68.18 Ω for P25 and P25-THSs) when THSs are introduced into the photoanode as a scattering layer. The largest *R*_2_ for P25-THSs is due to the highest dye adsorption density of P25-THSs. From the Bode phase plots of EIS spectra (Fig. 8b), we can obtain information on electron lifetime (*τ*_e_), and the specific data of *τ*_e_ could be estimated from the equation *τ*_e_ = 1/2π*f*, where *f* represents the characteristic frequencies of the maximum phase shift. As shown in Table 2, the *τ*_e_ of P25/MWNTs and P25/MWNTs-THSs DSSCs is 8.85 and 7.96 ms, respectively, which has been improved than for the DSSC without MWNTs under the same conditions. It should be a consequence of MWNTs providing a direct pathway for electron transport.

**Table 2:** EIS parameters of the DSSCs determined by fitting the experimental data to the equivalent circuit

DSSC types	*R*_s_ (Ω)	*R*_1_ (Ω)	*R*_2_ (Ω)	*τ*_e_ (ms)
P25	40.56	4.62	21.06	5.49
P25/MWNTs	43.36	4.46	20.46	8.85
P25-THSs	40.28	4.89	68.18	3.79
P25/MWNTs-THSs	43.88	4.07	22.01	7.96

## Conclusions

In summary, anatase THSs have been synthesized through a simple sacrifice template method. THSs, P25, and MWNTs were used in a composite photoanode. THSs were used as the scattering layer in the photoanode, enhancing the light harvesting; P25 mixed with MWNTs was used as the under-layer in the photoanode, not only absorbing dye molecules but also providing a rapid pathway for electron transfer. DSSC based on such a composite photoanode achieves an improved power conversion efficiency of 5.13 %, which is 14.25 % higher than that of P25-based DSSC (4.49 %). This composite photoanode will provide a new insight into the fabrication and structure design of highly efficient DSSCs.
